# Codon Usage in Signal Sequences Affects Protein Expression and Secretion Using Baculovirus/Insect Cell Expression System

**DOI:** 10.1371/journal.pone.0145887

**Published:** 2015-12-23

**Authors:** Yalan Wang, Yuanhui Mao, Xiaodong Xu, Shiheng Tao, Hongying Chen

**Affiliations:** 1 College of Life Sciences, Northwest A&F University, Yangling, Shaanxi 712100, P. R. China; 2 State key laboratory of crop stress biology for arid areas, Northwest A&F University, Yangling, Shaanxi 712100, P. R. China; Icahn School of Medicine at Mount Sinai, UNITED STATES

## Abstract

By introducing synonymous mutations into the coding sequences of GP64sp and FibHsp signal peptides, the influences of mRNA secondary structure and codon usage of signal sequences on protein expression and secretion were investigated using baculovirus/insect cell expression system. The results showed that mRNA structural stability of the signal sequences was not correlated with the protein production and secretion levels, and FibHsp was more tolerable to codon changes than GP64sp. Codon bias analyses revealed that codons for GP64sp were well de-optimized and contained more non-optimal codons than FibHsp. Synonymous mutations in GP64sp sufficiently increased its average codon usage frequency and resulted in dramatic reduction of the activity and secretion of luciferase. Protein degradation inhibition assay with MG-132 showed that higher codon usage frequency in the signal sequence increased the production as well as the degradation of luciferase protein, indicating that the synonymous codon substitutions in the signal sequence caused misfolding of luciferase instead of slowing down the protein production. Meanwhile, we found that introduction of more non-optimal codons into FibHsp could increase the production and secretion levels of luciferase, which suggested a new strategy to improve the production of secretory proteins in insect cells.

## Introduction

Synonymous codons refer to the different triplets which code for the same amino acid. All amino acids, except Met and Trp, are encoded by two to six codons. Increasing evidences have clearly shown that synonymous codons are not used equally in many species. This phenomenon is termed codon usage bias [1–5]. For instance, highly expressed genes in *Escherichia coli* and *Saccharomyces cerevisia*e have been found to selectively use optimal codons with high usage frequency [6, 7]. Considering that the genomic codon frequency is usually correlated with the abundance of the cognate tRNAs, the abundant tRNAs for their optimal codons can transport enough amino acids for the rapid decoding of these highly expressed genes by ribosomes. Therefore, a plausible explanation for the correlation of the usage of optimal codons with the production level of individual genes is that highly expressed genes are translated at higher speed than other genes [8]. Experiments have demonstrated that substitution of synonymous codons can affect protein translation level [9, 10], as well as other co-translational or post-translational processes including protein folding [8, 10–12], aggregation [11, 13], and translocation [12, 14].

Signal peptide, the short peptide which directs the newly synthesized protein to the secretory pathway in both prokaryotic and eukaryotic cells, majorly locates at the N terminus of secretory proteins. Although the sequences of signal peptides vary greatly, they all contain basic amino acids in the N-terminal region, followed by a middle hydrophobic core region and a C-terminal region containing polar amino acids. Different signal sequences can be interchanged between different proteins or even between proteins from different organisms. It is also a feasible way to improve the expression and secretion of some proteins by changing the type of the amino acids in their signal peptides [15–18]. Notably, genome-wide analyses have revealed high incidences of non-optimal codons at the N-terminal region of secretory protein genes in *E*. *coli* [19] and *Streptomyces coelicolor* [20]. Introducing synonymous codons with different usage frequency into signal peptides can provide new insights into the expression and secretion of secretory proteins. Using this strategy, it has been shown that non-optimal codons in the signal sequence of maltose binding protein (MBP) and β-lactamase are of great importance for the correct folding and export of the proteins [12, 21].

Codon substitutions are always coupled with the change of mRNA secondary structure, and the latter also acts as an important regulatory factor on gene expression. It is always hard to determine whether the codon usage or the mRNA structure is the determinant of protein expression and translocation. Generally, mRNA folding with higher free energy tends to have less secondary structure; conversely, that holding lower folding energy tends to form more stable secondary structure. Additional energy is required to unfold stable secondary structure, and this will obviously hinder ribosome from translation initiation or moving along the mRNA during protein synthesis [22, 23]. The stability of mRNA secondary structure adds complexity to protein expression regulation [24]. Recently, a stable mRNA secondary structure in the region of 30–80 nt downstream of the translation start codon was identified by computational analyses. The structural stability in this region was thought to be correlated with the translocation of secreted proteins [25–27]. This provides an interesting hypothesis that secondary structure at the N-terminus of the mRNAs for secretory proteins promotes ribosomal pause at early stage of elongation, benefitting the protein localization [26].

In this report, we investigated whether mRNA secondary structure stability or codon usage frequency in signal peptides affected protein expression and secretion using baculovirus/insect cell expression system. Two signal peptides, GP64sp from GP64 of *Autographa californica* multiple nucleopolyhedrovirus (AcMNPV) and FibHsp from the heavy chain of fibroin of *Bombyx mori*, were recoded with synonymous codons and fused to the firefly luciferase reporter gene. The expression and secretion of luciferase directed by these signal peptides were quantified and compared. We found that the correct folding and stability of the passenger protein was correlated with the non-optimal codon usage instead of the mRNA secondary structure stability in the signal sequences.

## Results

### Time course for the expression and secretion of luciferase

**Fig 1:**
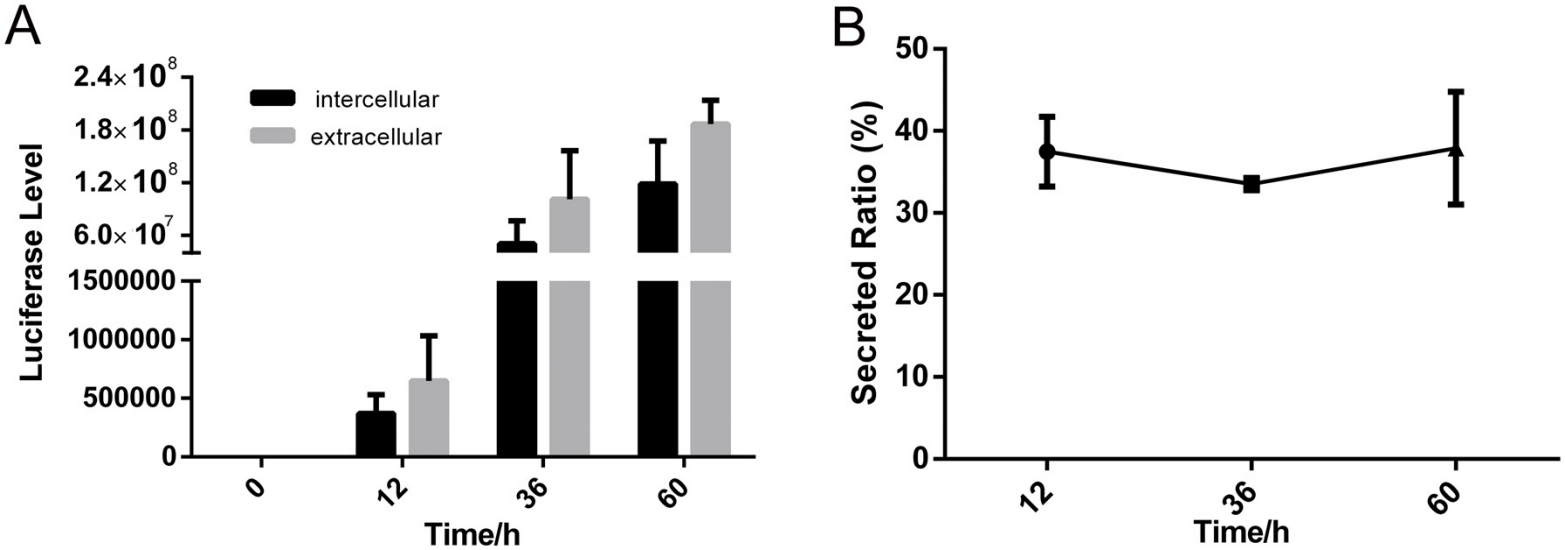
Time course for the expression and secretion of luciferase fused with FibHsp. A. Activity of the secreted (extracellular) and unsecreted (intracellular) luciferase. B. The ratios of secreted luciferase at the indicated time points.

To express firefly luciferase using baculovirus/insect cell expression system, the reporter’s gene was cloned into pBac-5, a vector containing modified gp64 tandem early and late promoters which can initiate protein expression immediately after infection and also drive continued protein expression in the late phase of infection. In a previous study, we have reported that the expression of fluorescent proteins under the control of these tandem promoters can be detected as early as 8 hours post-infection (hpi) [28]. Here, the expression and secretion levels of luciferase fused with FibHsp, a signal peptide of Fibroin heavy chain from *Bombyx mori*, were examined at 12, 36 and 60 hpi (Fig 1). The results showed that the enzyme activity of luciferase was detectable, but at low levels, in both *Sf*9 cells and the cell culture media at 12 hpi, and the protein levels increased dramatically at 36 hpi. Even though the protein expression levels increased about 30 fold from 12 hpi to 60 hpi, the average secretion ratios remained between 35% and 40%. Similar results were obtained for luciferase fused with signal peptide GP64sp (data not shown). Therefore, in all further studies in this report, the reporter protein was examined at the time point of 36 hpi, when the protein expression reached a reasonable high level and the virus-infected cells were still in good condition.

### Stability of mRNA secondary structure in GP64sp was not correlated with the protein expression and secretion

**Fig 2:**
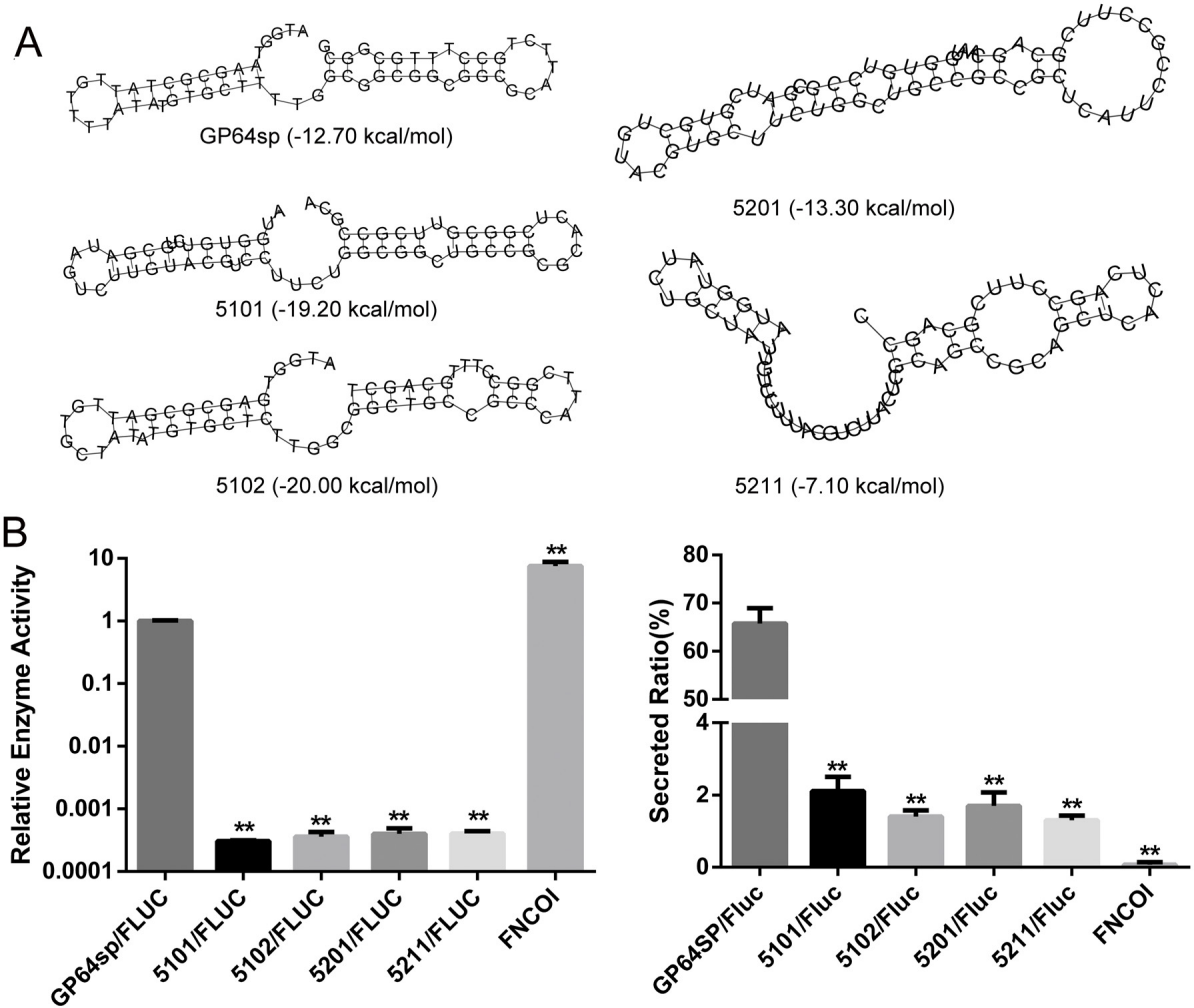
Expression and secretion of luciferase fused with GP64sp. A. The predicted mRNA secondary structure and minimal free energy for the indicated signal sequences. B. The expression and secretion levels of firefly luciferase with the indicated signal peptide. Intracellular and extracellular luciferase activities were separately detected, and the sum of the two was used to calculate the relative enzyme activity. **p<0.01 vs. the wild type signal sequence.

Secondary structure of mRNA has been considered to be an important factor that participates in translation regulations. In a previous report, we found a high structural stability region (HSR) in the 30–80 nt region of mRNAs encoding secretory proteins by bioinformatics [26]. Interestingly, a significant correlation between structural stability and protein localization was revealed based on genome analysis. In order to study whether the high stability of this region plays a role in protein expression and secretion, here, we introduced synonymous substitutions in *GP64sp* to acquire mutants with altered mRNA stability by computer. For each mutant, the minimal free energy (mfe), an indicator widely used in representing the secondary structure stability of mRNA, was calculated. Among all the possible mutants, *5101* and *5102* used in this study had the lowest mfe, corresponding to the reduced structural stability. *5201* had a middle mfe close to the wild type *GP64sp*, and *5211* had the highest mfe. The predicted mRNA secondary structure and mfe for *GP64sp* and its four mutants used in this study were shown in Fig 2A. The mutated signal sequences and wild type *GP64sp* were then fused to the upstream of *luciferase* gene to direct the secretion of luciferase. The luciferase activity of their secreted and non-secreted protein products was then examined in parallel with the enzyme expressed without signal peptide. As expected, luciferase without signal peptide (FNCOI in Fig 2B) was not secreted but produced in a much higher level than those with signal peptide, probably because the secretion process caused translation elongation arrest and slowed down the protein synthesis. Interestingly, in all of the four mutants, we found that both the enzyme activity and secretion ratio of the reporter decreased to very low levels, regardless of the mRNA structural stability (Fig 2B). Our results suggested that the stability of mRNA secondary structure in *GP64sp* had little effects on the expression and secretion of its passenger protein. Note that a strong codon usage bias was found in *GP64sp*, it is more possible that codon usage bias rather than structural stability of *GP64sp* affected the secretion and activity of the reporter protein.

### FibHsp is more stable than GP64sp in guiding protein expression and secretion

**Fig 3:**
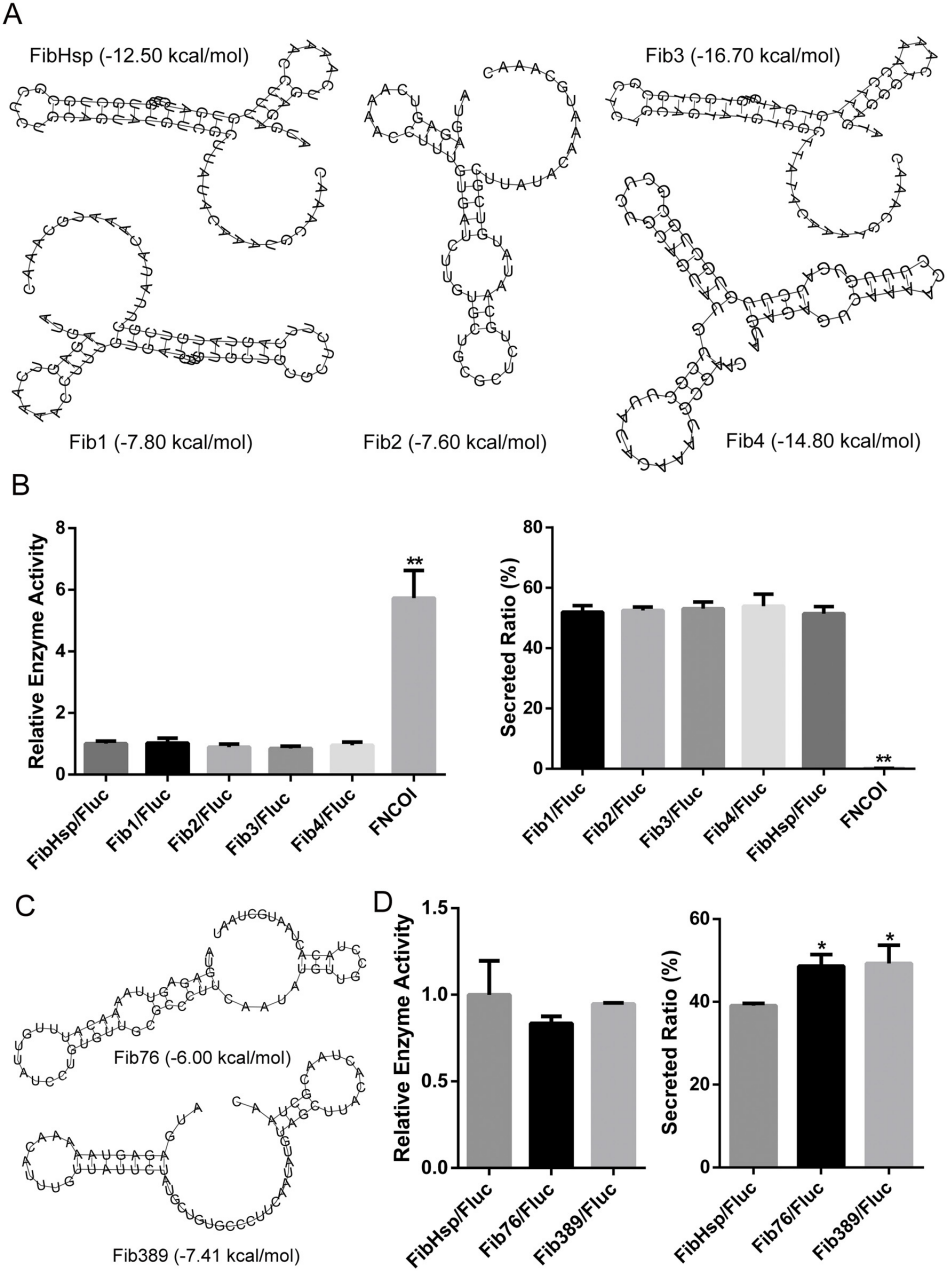
Expression and secretion of luciferase fused with FibHsp. A. The predicted mRNA secondary structure and minimal folding energy for *FibHsp* and its mutants containing single synonymous substitution. B. The expression and secretion levels of luciferase with the indicated signal peptide. C. The predicted mRNA secondary structure and minimal folding energy for FibHsp mutants containing multiple synonymous codon substitutions. D. The expression and secretion levels of luciferase fused with FibHsp mutants containing multiple synonymous codon substitutions. Intracellular and extracellular luciferase activities were separately detected, and the sum of the two was used to calculate the relative enzyme activity. *p< 0.05, **p<0.01 vs. the wild type signal sequence.

Containing 5 of 21 low-frequency codons (codon usage frequency <10 per thousand) in *GP64sp*, the dramatic reduction of luciferase activity and secretion ratio caused by the synonymous mutations implied that the codons for this signal sequence had been optimized during evolution. To further investigate the connection between the mRNA structural stability and protein expression and secretion, we introduced single synonymous codon substitutions into another signal sequence *FibHsp*. *FibHsp* contained less low-frequency codons than *GP64sp* and might be more tolerable to codon changes. To test this possibility, the mfe for every possible single *FibHsp* mutant was calculated. Among them, *Fib1* and *Fib2* with the highest mfe, and *Fib3* and *Fib4* with the lowest mfe were used to fuse with *luciferase* gene (Fig 3A). We found that the enzyme activity and secretion ratio of the single mutants were shown comparable to the wild type FibHsp (Fig 3B), confirming that mRNA secondary structure in signal peptide had no significant effect on protein expression and secretion.

Compared with the four GP64sp mutants, which possessed 11 to 17 synonymous codon substitutions and disrupted the reporter expression and secretion, single mutations in *FibHsp* caused comparable mfe changes but did not affect the reporter expression and secretion. To investigate whether this discrepancy was due to the difference of codon substitution numbers, two more *FibHsp* mutants, *Fib76* and *Fib389* which respectively contained 14 and 15 synonymous codon substitutions, folded into different secondary structures with less stability than *FibHsp* (Fig 3C), were obtained and fused to the *luciferase* gene. We found that the total enzyme activity including the intracellular and extracellular reporter led by the mutated signal peptides was expressed at similar levels with FibHsp, but the reporter’s secretion levels were moderately elevated by the mutated signal sequences (Fig 3D). The high protein expression and secretion levels under the direction of Fib76 and Fib389 further confirmed that protein expression and secretion were not correlated with the mRNA stability or structure of the signal sequence, and also suggested that FibHsp could function more stably than GP64sp.

### Synonymous mutations in *GP64sp* resulted in the inactivation of the reporter protein

**Fig 4:**
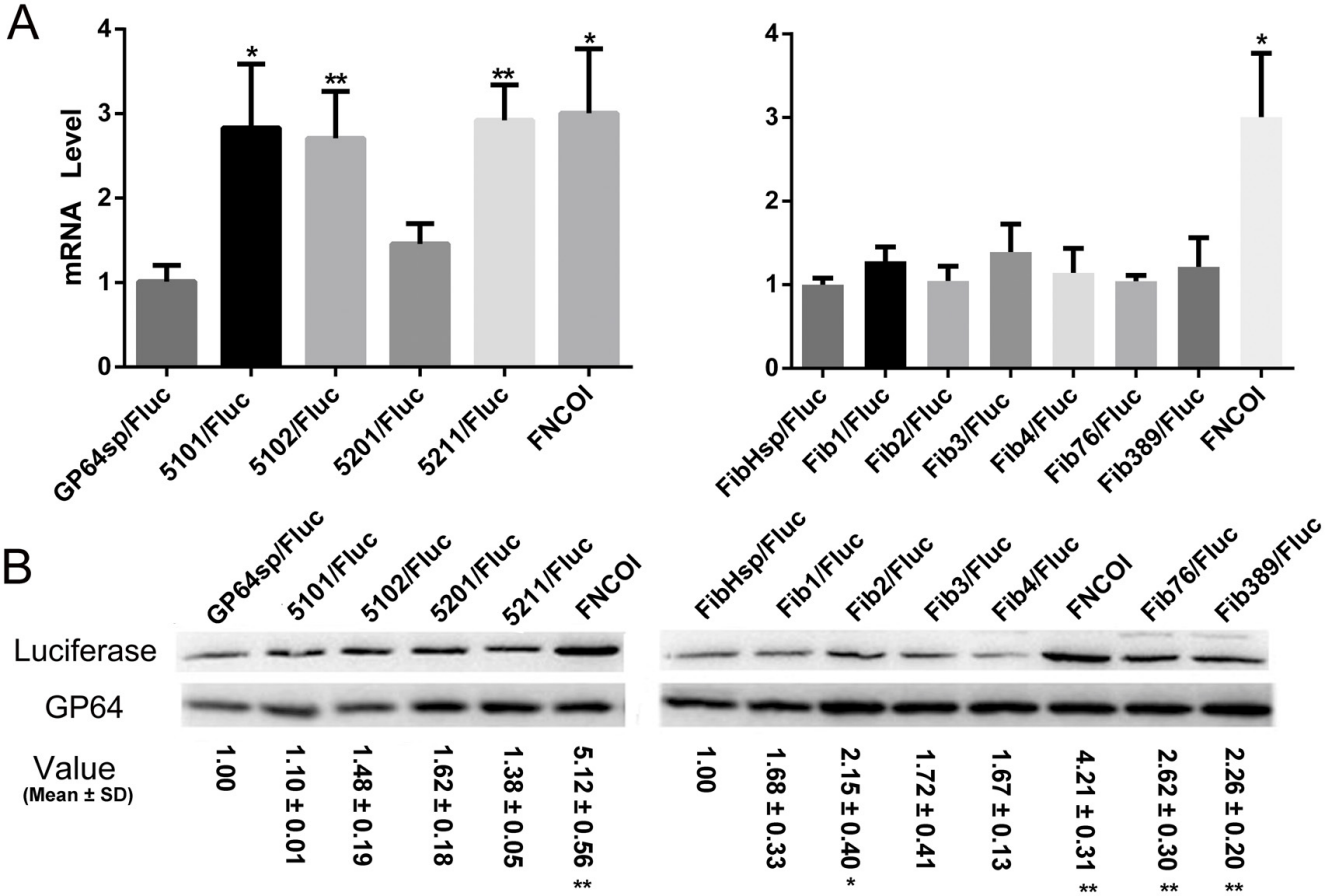
Transcriptional and translational levels of luciferase led by different signal sequences. A. Detection of the transcriptional levels of luciferase directed by different signal sequences by Real-time PCR. B. Western blot analyses of the translational levels of luciferase directed by different signal sequences. Luciferase protein was detected using anti-His monoclonal antibody, and baculovirus gp64 was also examined using anti-GP64 monoclonal antibody as an internal control for the baculovirus infection and sample loading. Relative expression levels of luciferase were normalized to GP64, and the value shown were representative of triplicated experiments. *p<0.05 and **p<0.01 vs. the wild type signal sequence.

To investigate whether the different reporter activities were regulated at the transcriptional level or translational level, real-time PCR and western blot were carried out to detect the mRNA and protein products of the reporter. Surprisingly, in the group of GP64sp, only the transcription level of 5201 was not significantly higher than the wild type, while the other three mutants obviously produced more mRNAs (Fig 4A). At the protein level, the mutants were also comparable with GP64sp (Fig 4B). As the enzyme activities of the mutants were shown to drop more than thousand times (Fig 2B), these results suggested that the synonymous codon substitutions in the signal sequence impaired luciferase function instead of slowing down the protein production.

As for the group of FibHsp, the single and multiple mutations did not result in significant changes of the mRNA production (Fig 4A). By western blot, most of the single mutants, except Fib2, were detected at similar levels with the wild type, but the multiple mutants FIB76 and FIB389 were found to be produced more abundantly than the other secreted proteins (Fig 4B). The increased production of protein directed by Fib76 and Fib389 may account for the strengthened secretion of the protein observed in Fig 3D.

### Non-optimal codons in the signal sequences benefited the secretion of luciferase

**Fig 5:**
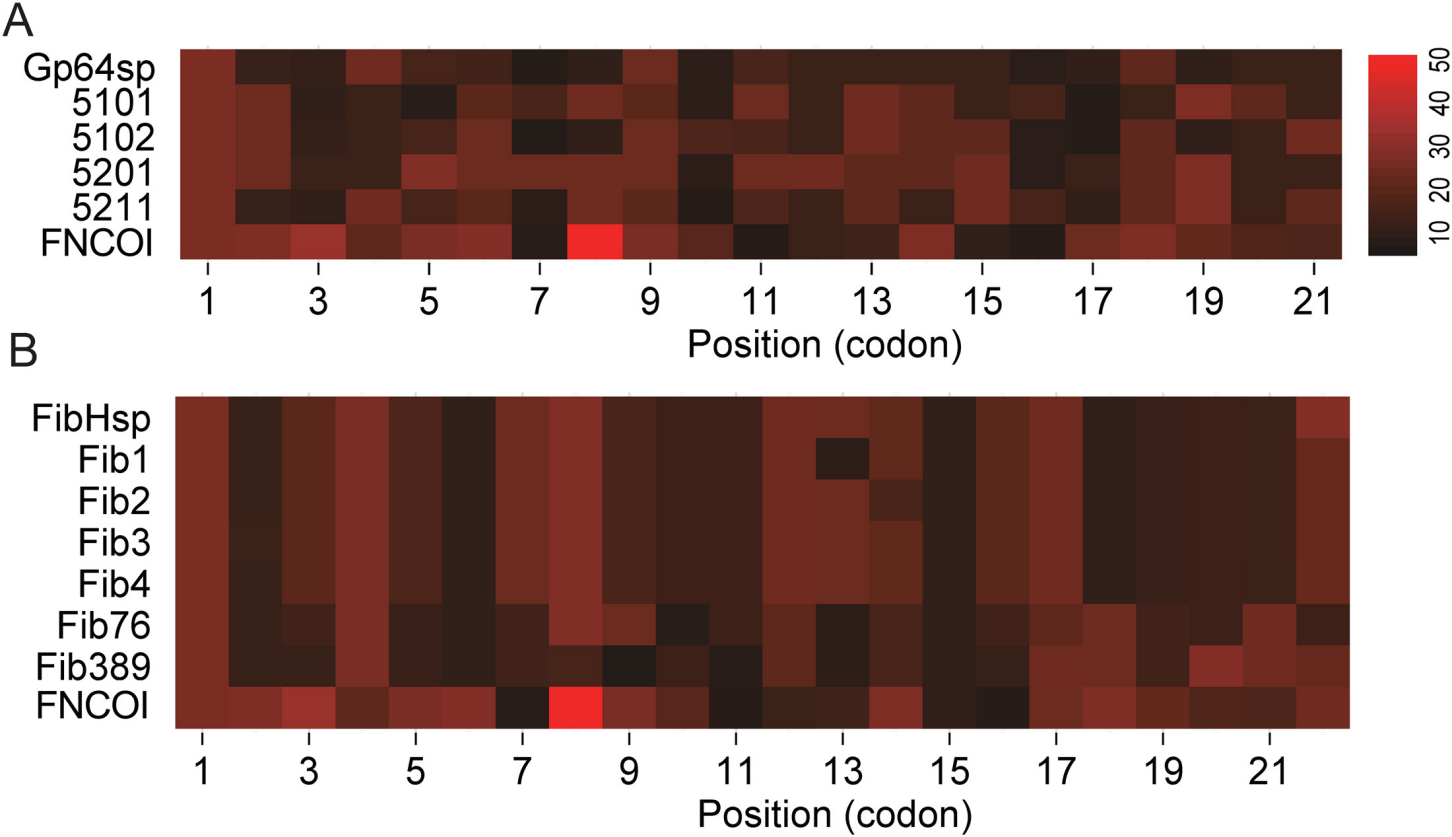
Codon usage frequency analysis. A. Codon usage frequency of GP64sp and its mutants. B. Codon usage frequency of FibHsp and its mutants. Numbers from 1 to 22 stand for the codon positions in the indicated signal sequences.

Previous reports have revealed that secreted proteins contain more non-optimal codons at their N-terminal region, and the usage of non-optimal codons in the signal sequence plays an important role for the correct folding and export of the secreted proteins in prokaryotic expression systems [12, 21]. To investigate the codon bias in the signal sequences used in this study and its connection with the protein production and secretion, codon usage frequencies of the codons in the signal sequences used in this study are listed in Fig 5 based on the data from *Spodoptera frugiperda* (http://www.kazusa.or.jp/codon/). The average codon frequency in both of the wild type signal sequences, especially *GP64sp*, are obviously lower than the first 21 or 22 codons for non-secreted luciferase (*FNCOI*), consisting with the observation of more non-optimal codons in secretory proteins in *E*. *coli* [19] and *Streptomyces coelicolor* [20].

In *GP64sp*, the codons are well de-optimized and the mutations in the four mutants sufficiently increased the average codon usage frequency in the signal sequences, and the number of codons used at the frequency higher than 20 are doubled or even tripled in the mutants. Less non-optimal codons in the mutants may speed up the translation of the protein but at the same time result in more mistakenly folded proteins, and this may contribute to the less enzyme activity detected in Fig 2 and explain why the protein abundant determined by western blot was not reduced in Fig 4.

For *FibHsp*, which contains 11 codons with usage frequency higher than 20 and 2 codons with minimum usage frequency of 10, the average codon usage frequency is obviously higher than *GP64sp* although it is still lower than *FNCOI*. In *Fib76* and *Fib389*, the mutations respectively introduced 3 more codons with usage frequency lower than 20 and 2 or 3 more codons with usage frequency lower than 10. The introduction of these non-optimal codons did not reduce the production of the protein but benefited the protein production (Fig 4B) and secretion (Fig 3D). The results obtained from both GP64sp and FibHsp groups suggested that non-optimal codons in the signal sequences could play an important role in the correct folding and export of the reporter protein.

### Luciferase directed by signal sequences with higher codon usage frequencies is more sensitive to the proteasome-dependent degradation

**Fig 6:**
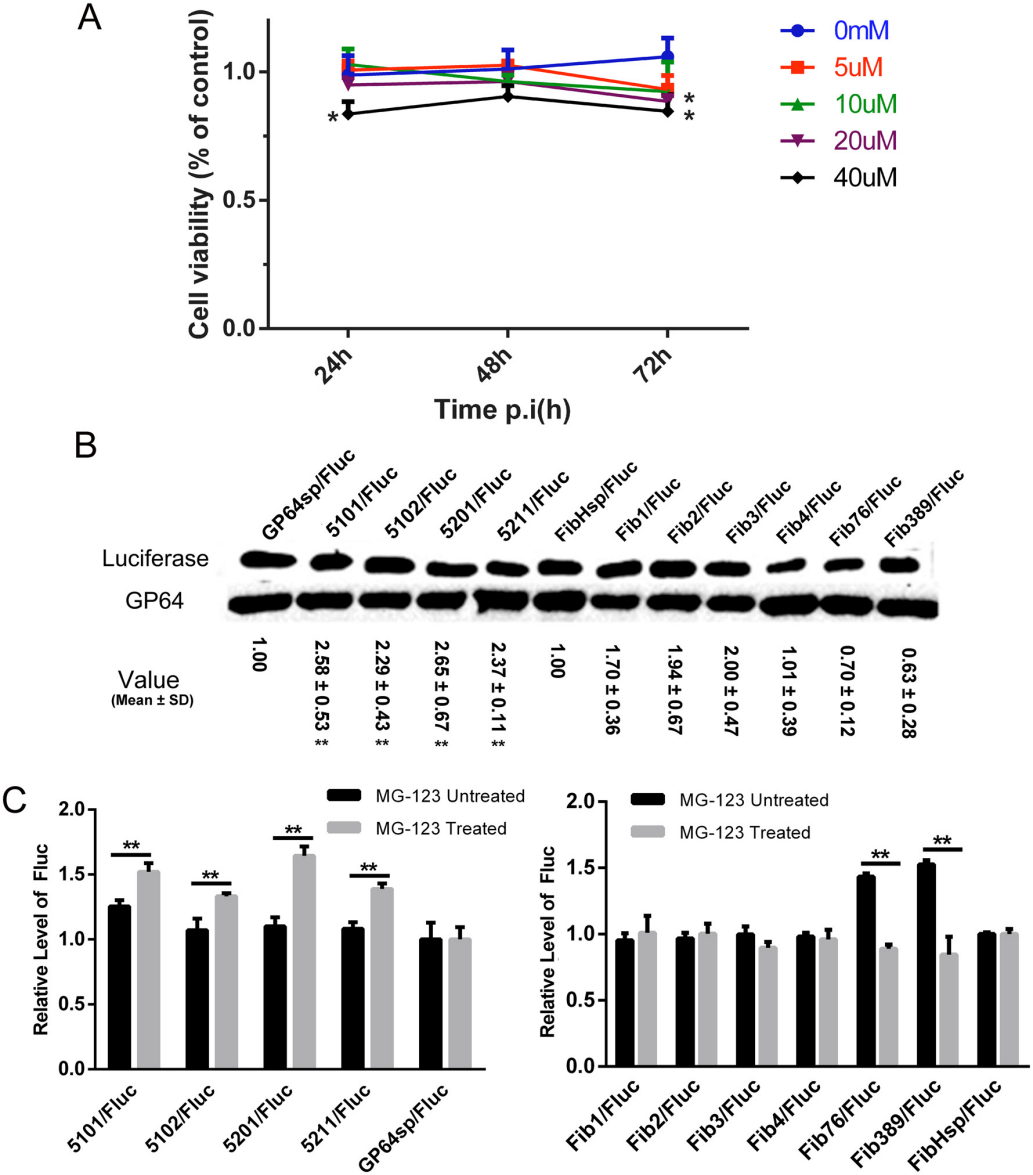
Protein expression levels with the treatment of MG-132. A. Drug toxicity assay. *Sf*9 cells were treated with MG-132 at the indicated concentrations and cell viability was determined by measuring succinate dehydrogenase level at intervals of 24 hours. B. Western blot analysis of the luciferase expression level with the treatment of 5μM MG-132. Luciferase protein was detected using anti-His monoclonal antibody, and baculovirus gp64 was also examined using anti-GP64 monoclonal antibody as an internal control for the baculovirus infection and sample loading. Relative expression levels of luciferase were normalized to GP64, and the relative value shown in the figure were representative of triplicated experiments. **p<0.01 vs. the wild type. C. Enzyme-linked immunosorbent assay for both MG-132 treated and untreated samples. **p<0.01, MG-132 treated vs. MG-132 untreated.

In Fig 2, luciferase activity was observed dramatically declined in the GP64sp mutants, suggesting that luciferase could be misfolded when it was directed by the mutated signal sequences containing more high-frequency codons. During protein synthesis, proteins that are unfolded or misfolded in the endoplasmic reticulum tend to be tagged with ubiquitin and then degraded in proteasome [29]. To investigate whether protein misfolding contributed to the decrease of luciferase activity directed by the *GP64sp* mutants, MG-132, a cell-permeable proteasome inhibitor widely used for reducing the degradation of ubiquitin-conjugated proteins in eukaryotic cells [30], was used in this study to inhibit misfolded luciferase from being degraded by proteasome. Several doses of MG-132 (5, 10, 20 and 40 μM) were assessed for the cytotoxicity of the drug. The cell viability assay showed that the doses at 5 and 10 μM did not statistically affect cell viability compared to the untreated control cells in 72 h of drug treatment (Fig 6A). To maintain sufficient cell viability for the baculovirus infection and protein expression, MG-132 was used at the concentration of 5 μM in subsequent protein degradation inhibition assays.

Western blot was then carried out to analyze luciferase expression level, which was normalized to the baculovirus protein GP64 in each infected sample (Fig 6B). By densitometric scanning of the bands from three independent experiments, the relative luciferase expression levels of the mutants were calculated to the protein level with the wild type signal peptide and shown in the figure. Given that Western blot is only a semi-quantitative method, Enzyme-linked immunosorbent assay (ELISA) was further carried out for more accurate measurement of the luciferase expression with or without MG-132 treatment (Fig 6C). The ELISA data were consistent with the Western blot results that the relative expression levels of luciferase fused with mutated GP64 signal peptide (5101, 5102, 5201 and 5211), significantly increased at the presence of MG-132. In the FibHsp group, Fib76 and Fib389, the two mutants containing more non-optimal codons and expressed at higher levels in the absence of MG-132, did not produce more proteins than FibHsp at the presence of MG-132. These results confirmed our postulation that more optimal codons in signal sequences could promote translation speed but result in more folding deficiency.

## Discussion

Using computational methods, we have found that mRNAs in the 30–80 nucleotide intervals for secretory proteins have significantly higher stability than other regions of secretory proteins and the region for non-secretory proteins [26]. In this study, by introducing synonymous codons into GP64sp and FibHsp, we investigated the influence of mRNA secondary structure stability of signal sequences on protein expression and secretion using baculovirus/insect cell expression system. The results show that mfe of HSR in GP64sp and FibHsp have no correlation with the protein expression and secretion, suggesting that the structural stability of the signal sequences is not the determinant for the production and translocation of secretory proteins although this structural region has undergone selection pressure to maintain high stability.

*GP64sp*, a signal sequence from *GP64* of baculovirus AcMNPV, has been widely used for the expression of secretory proteins in baculovirus/insect cell expression system. Here we find that this signal sequence has been well de-optimized and synonymous substitutions in this region could drastically affect the enzyme activity and secretion of luciferase as its passenger protein. By western blots, ELISA and protein degradation inhibition assay with MG-132, we show that introducing optimal codons in the signal sequence can increase the production as well as the degradation of luciferase protein. In prokaryotic cells, it has been shown that non-optimal codons in signal peptides play an important role in the correct folding of secretory MBP and β-lactamase [12, 21]. A reasonable explanation for these observations is that the non-optimal codons in the signal sequences may slowdown the translation elongation in this region and this could be important for the correctly folding and secretion of proteins.

Previous studies have suggested that optimized codons can contribute to the fast movement of ribosome, but it can also impair the activity of proteins or result in the proteolysis as the change of codon usage can affect the co-translational folding of protein [10, 31]. Data from synonymous substitution of signal sequences also suggest that high frequency usage of non-optimal codons in signal sequences probably plays a similar role in the regulation of translation in bacterial cells [12, 21]. The mechanism of protein translocation is still far from being completely understood. We speculate that non-optimal codons in signal sequences are required for the correct folding and binding of the nascent signal peptide to signal recognition particle. Misfolding of signal peptide caused by the fast decoding of the substituted higher frequency codons may interrupt the interaction of signal peptide with the signal recognition particle, and therefore block the on-going secretion and result in a disorder of the passenger protein translation. Further work is needed to verify this speculation.

Another interesting discovery from this study is that the codons for FibHsp, the signal peptide for the most abundant secretory protein in *Bombyx mori*, are not de-optimized as well as GP64sp and the sequence is more tolerable with synonymous codon substitutions. Introduction of non-optimal codons in Fib76 and Fib389 resulted in higher production and secretion levels of reporter protein. FibHsp is a potent signal peptide for the production of secreted proteins using baculovirus/insect cell expression platform. It is worthy to investigate whether introducing more non-optimal codons into FibHsp will further improve the production and secretion of passenger proteins.

To the best of our knowledge, this is the first study directly showing that synonymous codon substitutions of signal peptide have influence on passenger protein expression and secretion in eukaryotic cells. Further studies on codon bias in signal peptides may give us new insights into the protein co-translational process, including protein folding and translocation.

## Materials and Methods

### Secondary structure prediction

The prediction of mRNA secondary structure and calculation of minimum free energy (mfe) were achieved online using the website (http://rna.tbi.univie.ac.at/cgi-bin/RNAfold.cgi). Considering the optimal temperature for the growth of insect cell is 28°C, we set the parameter of temperature as 28°C, and other parameters were default settings.

### Plasmids Construction

The gene of Firefly luciferase, abbreviated as *Fluc*, was amplified from plasmid pGL3-Basic (Promega) by PCR, using primers FlucF and FlucR. *Fluc* was then cloned into vector pBac-5 (Novagen) between the *Hin*dIII and *Xho*I sites, and the generated construct was named as pBac-Fluc.

The gene sequences for wild type signal peptide of silkworm *Bombyx mori* fibroin heavy chain, *FibHsp*, and its mutants (*Fib1*, *Fib2*, *Fib3*, *Fib4*, *Fib76* and *Fib389*) were respectively generated by annealing and extension of oligonucleotides, using FibHspF and FibHspR, Fib1F and Fib1R, Fib1F and Fib2R, Fib3F and FibHspR, FibHspF and Fib4R, Fib76F and Fib76R, or Fib389F and Fib389R as primers. The gene sequences for wild type signal peptide of *Autographa californica* multiple nucleopolyhedrovirus (AcMNPV) gp64, *GP64sp*, was amplified from Bacmid BAC10:KO1629 [32]. *GP64sp* mutants (*5101*, *5102*, *5201* and *5211*) were respectively generated by annealing and extension of oligonucleotides, using gp64spF and gp64spR, 5102F and 5102R, 5201F and 5201R, or 5211F and 5211R as primers. All of the wild type and mutated gene fragments were cloned into pBac-Fluc vector between the *Nco*I and *Hin*dIII sites.

All the signal sequences used in this study are listed in Table 1. The sequences for the first 22 codons of FNCOI (the control with no signal peptide) are: ATG GAA GAC GCC AAA AAC ATA AAG AAA GGC CCG GCG CCA TTC TAT CCG CTG GAA GAT GGA ACC GCT.

**Table 1:** Signal sequence used in this study (mutated codons are underlined).

**Name**	**Sequence**
gp64sp	ATG GTA AGC GCT ATT GTT TTA TAT GTG CTT TTG GCG GCG GCG GCG CAT TCT GCC TTT GCG GCG
5101	ATG GTGTCTGCGATAGTCTTGTACGTC CTT CTG GCG GCTGCC GCG CACTCGGCGTTCGCCGCA
5102	ATG GTG AGC GCG ATT GTGCTA TAT GTG CTC TTG GCG GCTGCCGCC CAT TCG GCC TTT GCAGCT
5201	ATG GTGTCCGCGATCGTGCTGTAC GTG CTT CTGGCTGCCGCCGCT CAT TCC GCC TTCGCAGCA
5211	ATG GTA TCT GCT ATT GTCCTTTACGTCTTACTCGCAGCCGCAGCTCACTCA GCC TTCGCAGCC
FibHsp	ATG AGA GTC AAA ACC TTT GTG ATC TTG TGC TGC GCT CTG CAG TAT GTC GCT TAT ACA AAT GCA AAC
Fib1	ATG AGA GTC AAA ACC TTT GTG ATC TTG TGC TGC GCT CTT CAG TAT GTC GCT TAT ACA AAT GCA AAC
Fib2	ATG AGA GTC AAA ACC TTT GTG ATC TTG TGC TGC GCT CTG CAA TAT GTC GCT TAT ACA AAT GCA AAC
Fib3	ATG AGG GTC AAA ACC TTT GTG ATC TTG TGC TGC GCT CTG CAG TAT GTC GCT TAT ACA AAT GCA AAC
Fib4	ATG AGA GTC AAA ACC TTT GTG ATC TTG TGC TGC GCT CTG CAG TAT GTC GCT TAT ACA AAT GCG AAC
Fib76	ATG AGA GTT AAA ACA TTT GTT ATC CTGTGT TGC GCCCTTCAA TAT GTTGCCTACACT AAT GCTAAT
Fib389	ATG AGA GTA AAA ACA TTT GTTATTCTA TGC TGTGCCCTTCAA TAT GTA GCT TACACTAACGCT AAC

### Protein expression and detection

The transfer vectors were co-transfected with linearized BAC10:KO1629 DNA into *Spodoptera frugiperda* (*Sf*9) cells (Invitrogen) using Fugene HD Transfection Reagent (Roche). Cells were maintained at 27°C in SFX-INSECT medium (Thermo Scientific HyClone) with 1% fetal bovine serum (Thermo Scientific HyClone). Recombinant baculoviruses generated by homologous recombination were harvested at 5 days post transfection, and then *Sf*9 cells were infected with 100 μl of the culture medium containing recombinant baculoviruses.

For the detection of secreted luciferase, cell media were harvested at 12, 36 and/or 60 hours post infection (hpi). To detect the protein levels in *Sf*9 cells, cells in the plate were lysed by 5× cell lysis reagent (Promega) and then PBS was added to the same volume as the cell culture media. The enzyme activity of secreted (v) and unsecreted (w) firefly luciferase was determined by Glomax 20/20 Luminometer (Promega), using Luciferase Assay System (Promega). All of the samples were analyzed in triplicates, and each sample measurement was repeated three times. Secretion ratio (u) of the protein was calculated as below:

u=v/(v+w)×100%

### Western blot analysis

To detect the protein level in *Sf*9 cells, infected cells were harvested at 36 hpi and lysed with SDS loading buffer (2% SDS, 100 mM DTT, 0.1% bromophenol blue, 10% glycerol, 50 mM Tris-HCl pH 6.8). After boiling for 5 min, the cell lysates were analyzed by 12% SDS polyacrylamide gel electrophoresis (SDS-PAGE), transferred onto polyvinylidene difluoride (PVDF) membranes, and then blocked with 5% non-fat milk overnight at 4°C. The membranes were incubated, with anti-GP64 monoclonal antibody (Santa Cruz) for the normalization of protein samples, and with anti-His monoclonal antibody (CoWin Biotech, China) for the valuation of luciferase protein level, for 1 hour at room temperature. After three washes with TBS containing 0.1% Tween-20 (TBST), the membranes were incubated with HRP-conjugated goat anti-mouse antibody (CoWin Biotech, China) for 1 hour at room temperature, and washed again with TBST for three times. The proteins were then visualized by enhanced chemiluminescence using eECL Western Blot Kit (CoWin Biotech, China). Densitometry of Western blots was performed using Image Lab.

### Quantitative RT-PCR

To extract RNA, infected cells from a 6-well plate were lysed with 1 mL/well of TRIzon (Beijing CoWin Biotech). 5 ng of total RNA from each sample was subjected to reverse transcription, using PrimeScript 1st cDNA Synthesis Kit (Takara). Quantitative real time polymerase chain reaction (qPCR) was performed on CFX96 Real Time PCR System (BIO-RAD, USA), using SYBR Primix Ex Taq II (TaKaRa). To detect the transcription level of *luciferase*, specific forward primer (5′- CTGGAGACATAGCTTACTGGGACG -3′) and reverse primer (5′- GGTGTTGGAGCAAGATGGATTC -3′) were used for the qPCR. AcMNPV *gp64* mRNA, measured using specific forward primer (5′- TATGTGCTTTTGGCGGCGGC -3′) and reverse primer (5′- GCATACGCCTGGTAGTACCC -3′), was served as the internal reference for the total RNA level as well as the baculovirus infection efficiency. The reactions were carried out at 95°C for 10 min, followed by 40 cycles of 95°C for 10 s and 60°C for 30 s. Relative levels of *luciferase* mRNA were calculated using the 2^-ΔΔt^ method of relative quantification with *gp64*. All assays described here were repeated three times, and all of the measurements were made in triplicate.

### Cell viability assay

*Sf*9 cells were seeded in 96-well cell culture plates at approximately 3×10^4^ cells/well, and treated with 0, 5, 10, 20 or 40 μM MG-132 for 24 to 72 hours. Cell viability was analyzed by measuring the succinate dehydrogenase level with a MTT Cell Proliferation and Cytotoxicity Assay Kit (Beyotime, Beijing, PR China) according to the manufacturer's instruction.

### Protein degradation inhibition assay

*Sf*9 cells were seeded in 12-well cell culture plates and infected with recombinant baculoviruses. 5 μM MG-132 was added to block the proteolytic activity of proteasome complex 9, and the cells were harvested at 36 hpi. Western blots were then carried out to detect the expression levels of luciferase, using baculovirus GP64 as a control for the virus infection and protein loading. Densitometry of the signals was performed using Image Lab. Relative expression levels of luciferase were normalized to GP64. The results shown were representative of at least duplicated experiments.

### ELISA

*Sf*9 cells were seeded in 12-well plates and infected with recombinant baculoviruses expressing the luciferase. Cells infected with a baculovirus not expressing the luciferase were used for the baseline correction. 5 μM MG-132 was added to block the proteolytic activity of proteasome complex 9. The same volume of DMSO was added in parallel as the untreated control. The cells were harvested and lysed with cell lysis reagent (Promega) at 36 hpi. ELISA plates were coated with 30 μg/mL cell lysate diluted in 100 μL of 50 mM sodium carbonate buffer (pH 9.6) at 4°C overnight. The plates were washed three times with PBS containing 0.05% Tween-20 (PBST) and blocked with 5% nonfat milk in TBST buffer for 1 hour at 37°C. Anti-Luciferase polyclonal antibody (Promega) at the dilution of 1:250 was added and incubated at 37°C for 1 hour. After washing three times, 100 μL HRP-conjugated rabbit anti-goat IgG (diluted 1:2,000 in blocking buffer) was added to each well and incubated at 37°C for 1 hour. After washing with TBST, 50 μL of TMB was added and incubated in the dark at 37°C for 25 min. The reaction was stopped by adding 50 μL of 2M H_2_SO_4_, and the absorbance was read at 450 nm. All measurement was repeated three times.

## References

[1] Ikemura T. Codon usage and tRNA content in unicellular and multicellular organisms. Molecular biology and evolution. 1985;2(1):13–34. 3916708 10.1093/oxfordjournals.molbev.a040335

[2] Plotkin JB, Kudla G. Synonymous but not the same: the causes and consequences of codon bias. Nature Reviews Genetics. 2011;12(1):32–42. 10.1038/nrg2899 21102527 PMC3074964

[3] Drummond DA, Wilke CO. Mistranslation-induced protein misfolding as a dominant constraint on coding-sequence evolution. Cell. 2008;134(2):341–52. 10.1016/j.cell.2008.05.042 18662548 PMC2696314

[4] Sharp PM, Cowe E, Higgins DG, Shields DC, Wolfe KH, Wright F. Codon usage patterns in Escherichia coli, Bacillus subtilis, Saccharomyces cerevisiae, Schizosaccharomyces pombe, Drosophila melanogaster and Homo sapiens; a review of the considerable within-species diversity. Nucleic Acids Research. 1988;16(17):8207–11. 3138659 10.1093/nar/16.17.8207 PMC338553

[5] Akashi H. Synonymous codon usage in Drosophila melanogaster: natural selection and translational accuracy. Genetics. 1994;136(3):927–35. 8005445 10.1093/genetics/136.3.927 PMC1205897

[6] Ikemura T. Correlation between the abundance of Escherichia coli transfer RNAs and the occurrence of the respective codons in its protein genes: a proposal for a synonymous codon choice that is optimal for the E. coli translational system. Journal of molecular biology. 1981;151(3):389–409. 6175758 10.1016/0022-2836(81)90003-6

[7] Ikemura T. Correlation between the abundance of yeast transfer RNAs and the occurrence of the respective codons in protein genes: differences in synonymous codon choice patterns of yeast and Escherichia coli with reference to the abundance of isoaccepting transfer RNAs. Journal of molecular biology. 1982;158(4):573–97. 6750137 10.1016/0022-2836(82)90250-9

[8] Spencer PS, Siller E, Anderson JF, Barral JM. Silent substitutions predictably alter translation elongation rates and protein folding efficiencies. Journal of molecular biology. 2012;422(3):328–35. 10.1016/j.jmb.2012.06.010 22705285 PMC3576719

[9] Lakey DL, Voladri RK, Edwards KM, Hager C, Samten B, Wallis RS, et al Enhanced production of recombinant Mycobacterium tuberculosis antigens in Escherichia coli by replacement of low-usage codons. Infection and immunity. 2000;68(1):233–8. 10603393 10.1128/iai.68.1.233-238.2000 PMC97126

[10] Zhou M, Guo J, Cha J, Chae M, Chen S, Barral JM, et al Non-optimal codon usage affects expression, structure and function of clock protein FRQ. Nature. 2013 3 7;495(7439):111–5. Pubmed Central PMCID: 3629845. 10.1038/nature11833 23417067 PMC3629845

[11] Cortazzo P, Cerveñansky C, Marín M, Reiss C, Ehrlich R, Deana A. Silent mutations affect in vivo protein folding in Escherichia coli. Biochemical and biophysical research communications. 2002;293(1):537–41. 12054634 10.1016/S0006-291X(02)00226-7

[12] Zalucki YM, Jones CE, Ng PS, Schulz BL, Jennings MP. Signal sequence non-optimal codons are required for the correct folding of mature maltose binding protein. Biochimica et Biophysica Acta (BBA)-Biomembranes. 2010;1798(6):1244–9. 20230779 10.1016/j.bbamem.2010.03.010

[13] Fedyunin I, Lehnhardt L, Böhmer N, Kaufmann P, Zhang G, Ignatova Z. tRNA concentration fine tunes protein solubility. FEBS letters. 2012;586(19):3336–40. 10.1016/j.febslet.2012.07.012 22819830

[14] Ng DT, Sarkar CA. Engineering signal peptides for enhanced protein secretion from Lactococcus lactis. Applied and environmental microbiology. 2013;79(1):347–56. 10.1128/AEM.02667-12 23124224 PMC3536070

[15] Futatsumori-Sugai M, Tsumoto K. Signal peptide design for improving recombinant protein secretion in the baculovirus expression vector system. Biochemical and biophysical research communications. 2010;391(1):931–5. 10.1016/j.bbrc.2009.11.167 19962965

[16] Tsuchiya Y, Morioka K, Shirai J, Yokomizo Y, Yoshida K, editors. Gene design of signal sequence for effective secretion of protein Nucleic acids symposium series; 2003: Oxford Univ Press. 10.1093/nass/3.1.261 14510480

[17] Tsuchiya Y, Morioka K, Shirai J, Yoshida K, editors. Structural requirements of signal peptide in insect cell Nucleic Acids Symposium Series; 2004: Oxford Univ Press. 10.1093/nass/48.1.181 17150538

[18] Tsuchiya Y, Morioka K, Taneda I, Shirai J, Yoshida K, editors. Gene design of signal sequence for the effective secretion of recombinant protein using insect cell Nucleic Acids Symposium Series; 2005: Oxford Univ Press. 10.1093/nass/49.1.305 17150755

[19] Power PM, Jones RA, Beacham IR, Bucholtz C, Jennings MP. Whole genome analysis reveals a high incidence of non-optimal codons in secretory signal sequences of Escherichia coli. Biochemical and biophysical research communications. 2004;322(3):1038–44. 15336569 10.1016/j.bbrc.2004.08.022

[20] Li Y-D, Li Y-Q, Chen J-s, Dong H-j, Guan W-J, Zhou H. Whole genome analysis of non-optimal codon usage in secretory signal sequences of Streptomyces coelicolor. Biosystems. 2006;85(3):225–30. 16644095 10.1016/j.biosystems.2006.02.006

[21] Zalucki YM, Gittins KL, Jennings MP. Secretory signal sequence non-optimal codons are required for expression and export of β-lactamase. Biochemical and biophysical research communications. 2008;366(1):135–41. 18053805 10.1016/j.bbrc.2007.11.093

[22] Kudla G, Murray AW, Tollervey D, Plotkin JB. Coding-sequence determinants of gene expression in Escherichia coli. science. 2009;324(5924):255–8. 10.1126/science.1170160 19359587 PMC3902468

[23] Wen J-D, Lancaster L, Hodges C, Zeri A-C, Yoshimura SH, Noller HF, et al Following translation by single ribosomes one codon at a time. Nature. 2008;452(7187):598–603. 10.1038/nature06716 18327250 PMC2556548

[24] Wan Y, Kertesz M, Spitale RC, Segal E, Chang HY. Understanding the transcriptome through RNA structure. Nature Reviews Genetics. 2011;12(9):641–55. 10.1038/nrg3049 21850044 PMC3858389

[25] Pop C, Rouskin S, Ingolia NT, Han L, Phizicky EM, Weissman JS, et al Causal signals between codon bias, mRNA structure, and the efficiency of translation and elongation. Mol Syst Biol. 2014;10:770 Pubmed Central PMCID: 4300493. 10.15252/msb.20145524 25538139 PMC4300493

[26] Mao Y, Wang W, Cheng N, Li Q, Tao S. Universally increased mRNA stability downstream of the translation initiation site in eukaryotes and prokaryotes. Gene. 2013;517(2):230–5. 10.1016/j.gene.2012.12.062 23313297

[27] Fluman N, Navon S, Bibi E, Pilpel Y. mRNA-programmed translation pauses in the targeting of E. coli membrane proteins. eLife. 2014;3 . Pubmed Central PMCID: 4359368. 25135940 10.7554/eLife.03440 PMC4359368

[28] Xu X, Chen Y, Zhao Y, Liu X, Dong B, Jones IM, et al Baculovirus superinfection: a probable restriction factor on the surface display of proteins for library screening. PloS one. 2013;8(1):e54631 10.1371/journal.pone.0054631 23365677 PMC3554712

[29] Kostova K, Zlatka, Wolf DH. For whom the bell tolls: protein quality control of the endoplasmic reticulum and the ubiquitin–proteasome connection. The EMBO journal. 2003;22(10):2309–17. 12743025 10.1093/emboj/cdg227 PMC155985

[30] Lee DH, Goldberg AL. Proteasome inhibitors: valuable new tools for cell biologists. Trends in cell biology. 1998;8(10):397–403. 9789328 10.1016/s0962-8924(98)01346-4

[31] Siller E, DeZwaan DC, Anderson JF, Freeman BC, Barral JM. Slowing bacterial translation speed enhances eukaryotic protein folding efficiency. Journal of molecular biology. 2010 3 12;396(5):1310–8. 10.1016/j.jmb.2009.12.042 20043920

[32] Zhao Y, Chapman DA, Jones IM. Improving baculovirus recombination. Nucleic acids research. 2003;31(2):e6–e. 12527795 10.1093/nar/gng006 PMC140531

